# The influence of obesity on survival in early, high-risk breast cancer: results from the randomized SUCCESS A trial

**DOI:** 10.1186/s13058-015-0639-3

**Published:** 2015-09-18

**Authors:** Peter Widschwendter, Thomas WP Friedl, Lukas Schwentner, Nikolaus DeGregorio, Bernadette Jaeger, Amelie Schramm, Inga Bekes, Miriam Deniz, Krisztian Lato, Tobias Weissenbacher, Bernd Kost, Ulrich Andergassen, Julia Jueckstock, Julia Neugebauer, Elisabeth Trapp, Peter A. Fasching, Matthias W. Beckmann, Andreas Schneeweiss, Ines Schrader, Brigitte Rack, Wolfgang Janni, Christoph Scholz

**Affiliations:** Department of Gynecology and Obstetrics, University Ulm, Prittwitzstraße 43, 89075 Ulm, Germany; Department of Gynecology and Obstetrics, Campus Innenstadt Ludwig Maximilian University Munich, Professor Huber Platz 2, 80539 Munich, Germany; Department of Gynecology and Obstetrics, University Hospital Erlangen, Friedrich-Alexander University Erlangen-Nuremberg, Comprehensive Cancer Center Erlangen-EMN, Schloßplatz 4, 91054 Erlangen, Germany; Department of Gynecology and Obstetrics, University of Heidelberg, Grabengasse 1, 69117 Heidelberg, Germany; Gynecologic-Oncological Office, Pelikanplatz 23, 30177 Hannover, Germany

## Abstract

**Introduction:**

Obese breast cancer patients have worse prognosis than normal weight patients, but the level at which obesity is prognostically unfavorable is unclear.

**Methods:**

This retrospective analysis was performed using data from the SUCCESS A trial, in which 3754 patients with high-risk early breast cancer were randomized to anthracycline- and taxane-based chemotherapy with or without gemcitabine. Patients were classified as underweight/normal weight (body mass index (BMI) < 25.0), overweight (BMI 25.0–29.9), slightly obese (BMI 30.0–34.9), moderately obese (BMI 35.0–39.9) and severely obese (BMI ≥ 40.0), and the effect of BMI on disease-free survival (DFS) and overall survival (OS) was evaluated (median follow-up 65 months). In addition, subgroup analyses were conducted to assess the effect of BMI in luminal A-like, luminal B-like, HER2 (human epidermal growth factor 2)-positive and triple-negative tumors.

**Results:**

Multivariate analyses revealed an independent prognostic effect of BMI on DFS (*p* = 0.001) and OS (*p* = 0.005). Compared with underweight/normal weight patients, severely obese patients had worse DFS (hazard ratio (HR) 2.70, 95 % confidence interval (CI) 1.71–4.28, *p* < 0.001) and OS (HR 2.79, 95 % CI 1.63–4.77, *p* < 0.001), while moderately obese, slightly obese and overweight patients did not differ from underweight/normal weight patients with regard to DFS or OS. Subgroup analyses showed a similar significant effect of BMI on DFS and OS in patients with triple-negative breast cancer (TNBC), but not in patients with other tumor subtypes.

**Conclusions:**

Severe obesity (BMI ≥ 40) significantly worsens prognosis in early breast cancer patients, particularly for triple-negative tumors.

**Trial registration:**

Clinicaltrials.gov NCT02181101. Registered September 2005.

## Introduction

Obesity is a major risk factor for morbidity throughout the world [1]. It is associated with an increased risk of mortality attributable to diabetes, kidney diseases, coronary heart disease and other cardiovascular ailments [2]. From 1980 to 2013, the prevalence of obesity and overweight in adults worldwide increased by 27.5 %, with 2.1 billion people having a body mass index (BMI) greater than 25.

Several studies have shown that BMI influences the outcomes of breast cancer patients [3–6]; however, most of these studies categorized all patients with BMI ≥ 30 as obese. In a recent pooled analysis of four randomized clinical trials (5683 patients), Pajares et al. defined different levels of obesity (30–35 and > 35), and found that patients with BMI > 35 had a significantly higher risk for recurrence than did patients with BMI < 25, but patients with BMI 30–35 had similar prognosis as the reference group (BMI < 25) [7]. This indicates that more research is needed to evaluate the exact association between obesity and breast cancer outcomes, in particular with respect to the level of obesity at which there is an increased risk of breast cancer recurrence. We therefore conducted a retrospective analysis of the effect of BMI on outcomes in breast cancer patients treated with anthracycline- and taxane-based chemotherapy within the prospective randomized SUCCESS A trial. In addition, we performed subgroup analyses to evaluate whether the effect of BMI varied by tumor subtype.

## Methods

### Study design and patients

In the open-label, phase III clinical trial SUCCESS A (EudraCT 2005-000490-21), patients with high-risk breast cancer (histopathological proof of axillary lymph node metastases (pN1–3) or high-risk node-negative breast cancer defined as tumor size ≥ pT2, histological grade 3, negative hormone receptor status or age ≤ 35 years) were randomized to adjuvant chemotherapy treatment with three cycles of epirubicin, fluorouracil and cyclophosphamide (FEC, 500/100/500 mg/m^2^, every 3 weeks) followed by either three cycles of docetaxel (Doc, 100 mg/m^2^, every 3 weeks) or three cycles of docetaxel and gemcitabine (DocG, docetaxel 75 mg/m^2^ every 3 weeks plus gemcitabine 1000 mg/m^2^ day 1 and 8 every 3 weeks). After the end of chemotherapy, patients with hormone receptor-positive disease received tamoxifen for 2 years. Premenopausal patients continued tamoxifen treatment for an additional 3 years, while postmenopausal patients switched to anastrozole treatment for 3 years. Inclusion and exclusion criteria as well as therapeutic details are published elsewhere [8]. Body weight and height were measured before the start of chemotherapy, and BMI was calculated as weight in kilograms divided by the square of height in meters [9]. Patients were classified as underweight (BMI < 18.5 kg/m^2^; n = 45), normal weight (BMI 18.5–24.9 kg/m^2^; n = 1713), overweight (BMI 25.0–29.9 kg/m^2^; n = 1208), slightly obese (BMI 30.0–34.9 kg/m^2^; n = 554), moderately obese (BMI 35.0–39.9 kg/m^2^; n = 177) or severely obese (BMI ≥ 40.0 kg/m^2^; n = 57). For all analyses, underweight and normal weight patients were combined into one group (underweight/normal weight, n = 1758) because of the low number of patients with a BMI below 18.5 kg/m^2^. Treatment, therapy and monitoring were in accordance with the statutory provisions determined by the involved ethics committees of the Ludwig Maximilians University Munich, the German Federal Institute for Drugs and Medical Devices (BfArM) and good clinical practice (GCP). The trial was approved by the ethics committee of the medical faculty of the Ludwig Maximilians University (project number: 076–05; EudraCT 2005-000490-21). All patients gave written informed consent for participating in the SUCCESS A trial.

In total, 3754 patients at 271 study centers were randomized for the SUCCESS A trial between September 2005 and March 2007. Patient and tumor characteristics of the patients are shown in Table 1.

**Table 1:** Distribution of patient and tumor characteristics by body mass index (BMI) group

		Total (n = 3754)	BMI (kg/m^2^)					*p* value^a^
			<25.0	25.0–29.9	30.0–34.9	35.0–39.9	≥40.0	
			Underweight/normal weight	Overweight	Slightly obese	Moderately obese	Severely obese	
			(n = 1758)	(n = 1208)	(n = 554)	(n = 177)	(n = 57)	
Age (years)	Median	53.0	50.0	56.0	58.0	58.0	54.0	<0.001^b^
	Range	21–86	21–86	22–79	32–77	33–74	29–74	
Menopausal status	Premenopausal	1565 (41.7 %)	932 (53.0 %)	410 (33.9 %)	153 (27.6 %)	52 (29.4 %)	18 (31.6 %)	<0.001^c^
	Postmenopausal	2189 (58.3 %)	826 (47.0 %)	798 (66.1 %)	401 (72.4 %)	125 (70.6 %)	39 (68.4 %)	
Tumor size	pT1	1552 (41.3 %)	866 (49.3 %)	455 (37.7 %)	161 (29.1 %)	56 (31.6 %)	14 (24.6 %)	<0.001^d^
	pT2	1929 (51.4 %)	785 (44.7 %)	666 (55.1 %)	342 (61.7 %)	101 (57.1 %)	35 (61.4 %)	
	pT3	198 (5.3 %)	73 (4.2 %)	70 (5.8 %)	35 (6.3 %)	14 (7.9 %)	6 (10.5 %)	
	pT4	52 (1.4 %)	18 (1.0 %)	13 (1.1 %)	13 (2.3 %)	6 (3.4 %)	2 (3.5 %)	
	Unknown	23 (0.6 %)	16 (0.9 %)	4 (0.3 %)	3 (0.5 %)	0 (0.0 %)	0 (0.0 %)	
Nodal stage	pN0	1273 (33.9 %)	660 (37.5 %)	370 (30.6 %)	153 (27.6 %)	67 (37.9 %)	23 (40.4 %)	<0.001^d^
	pN1	1705 (45.4 %)	789 (44.9 %)	564 (46.7 %)	265 (47.8 %)	68 (38.4 %)	19 (33.3 %)	
	pN2	511 (13.6 %)	212 (12.1 %)	174 (14.4 %)	94 (17.0 %)	24 (13.6 %)	7 (12.3 %)	
	pN3	236 (6.3 %)	76 (4.3 %)	94 (7.8 %)	40 (7.2 %)	18 (10.2 %)	8 (14.0 %)	
	Unknown	29 (0.8 %)	21 (1.2 %)	6 (0.5 %)	2 (0.4 %)	0 (0.0 %)	0 (0.0 %)	
Histological grading	G1	176 (4.7 %)	81 (4.6 %)	59 (4.9 %)	27 (4.9 %)	8 (4.5 %)	1 (1.8 %)	0.126^d^
	G2	1783 (47.5 %)	848 (48.2 %)	576 (47.7 %)	255 (46.0 %)	82 (46.3 %)	22 (38.6 %)	
	G3	1773 (47.2 %)	814 (46.3 %)	568 (47.0 %)	270 (48.7 %)	87 (49.2 %)	34 (59.6 %)	
	Unknown	22 (0.6 %)	15 (0.9 %)	5 (0.4 %)	2 (0.4 %)	0 (0.0 %)	0 (0.0 %)	
Histological type	Invasive ductal	3060 (81.5 %)	1434 (81.6 %)	992 (82.1 %)	435 (78.5 %)	148 (83.6 %)	51 (89.5 %)	0.337^c^
	Invasive lobular	419 (11.2 %)	188 (10.7 %)	138 (11.4 %)	72 (13.0 %)	19 (10.7 %)	2 (3.5 %)	
	Other	253 (6.7 %)	121 (6.9 %)	73 (6.0 %)	45 (8.1 %)	10 (5.6 %)	4 (7.0 %)	
	Unknown	22 (0.6 %)	15 (0.9 %)	5 (0.4 %)	2 (0.4 %)	0 (0.0 %)	0 (0.0 %)	
Hormone receptor status	Negative	1100 (29.3 %)	531 (30.2 %)	337 (27.9 %)	163 (29.4 %)	44 (24.9 %)	25 (43.9 %)	0.049^c^
	Positive	2633 (70.1 %)	1212 (68.9 %)	866 (71.7 %)	390 (70.4 %)	133 (75.1 %)	32 (56.1 %)	
	Unknown	21 (0.6 %)	15 (0.9 %)	5 (0.4 %)	1 (0.2 %)	0 (0.0 %)	0 (0.0 %)	
HER2 status	Negative	2787 (74.2 %)	1304 (74.2 %)	887 (73.4 %)	417 (75.3 %)	135 (76.3 %)	44 (77.2 %)	0.838^c^
	Positive	883 (23.5 %)	418 (23.8 %)	292 (24.2 %)	122 (22.0 %)	39 (22.0 %)	12 (21.1 %)	
	Unknown	84 (2.2 %)	36 (2.0 %)	29 (2.4 %)	15 (2.7 %)	3 (1.7 %)	1 (1.8 %)	
Molecular subtype	Luminal A like	1426 (38.0 %)	673 (38.3 %)	454 (37.6 %)	222 (40.1 %)	63 (35.6 %)	14 (24.6 %)	0.050^c^
	Luminal B like	619 (16.5 %)	262 (14.9 %)	214 (17.7 %)	93 (16.8 %)	39 (22.0 %)	11 (19.3 %)	
	HER2 positive	883 (23.5 %)	418 (23.8 %)	292 (24.2 %)	122 (22.0 %)	39 (22.0 %)	12 (21.1 %)	
	Triple negative	742 (19.8 %)	369 (21.0 %)	219 (18.1 %)	102 (18.4 %)	33 (18.6 %)	19 (33.3 %)	
	Unknown	84 (2.2 %)	36 (2.0 %)	29 (2.4 %)	15 (2.7 %)	3 (1.7 %)	1 (1.8 %)	
Type of surgery	Breast conserving	2638 (70.3 %)	1205 (68.5 %)	859 (71.1 %)	399 (72.0 %)	135 (76.3 %)	40 (70.2 %)	0.229^c^
	Mastectomy	1097 (29.2 %)	539 (30.7 %)	345 (28.6 %)	154 (27.8 %)	42 (23.7 %)	17 (29.8 %)	
	Unknown	19 (0.5 %)	14 (0.8 %)	4 (0.3 %)	1 (0.2 %)	0 (0.0 %)	0 (0.0 %)	
Chemotherapy	FEC-DocG	1856 (49.4 %)	865 (49.2 %)	614 (50.8 %)	261 (47.1 %)	85 (48.0 %)	31 (54.4 %)	0.580^c^
	FEC-Doc	1898 (50.6 %)	893 (50.8 %)	594 (49.2 %)	293 (52.9 %)	92 (52.0 %)	26 (45.6 %)	
Undertreatment	Yes (<6 cycles CT)	359 (9.6 %)	149 (8.5 %)	113 (9.4 %)	64 (11.6 %)	24 (13.6 %)	9 (15.8 %)	0.029^c^
	No (6 cycles CT)	3395 (90.4 %)	1609 (91.5 %)	1095 (90.6 %)	490 (88.4 %)	153 (86.4 %)	48 (84.2 %)	
Antihormone therapy	No	1048 (27.9 %)	500 (28.4 %)	331 (27.4 %)	149 (26.9 %)	43 (24.3 %)	25 (43.9 %)	0.061^c^
	Yes	2677 (71.3 %)	1242 (70.6 %)	868 (71.9 %)	401 (72.4 %)	134 (75.7 %)	32 (56.1 %)	
	Unknown	29 (0.8 %)	16 (0.9 %)	9 (0.7 %)	4 (0.7 %)	0 (0.0 %)	0 (0.0 %)	

*HER2* human epidermal growth factor receptor 2, *FEC-DocG* 5-fluorouracile, epirubicine, cyclophosphamide – docetaxel, gemcitabine, *FEC-Doc* 5-fluorouracile, epirubicine, cyclophosphamide – docetaxel, *CT* chemotherapy

^a^All tests without category “unknown”

^b^Kruskal-Wallis test

^c^Chi-square test

^d^Mantel-Haenszel linear-by-linear association chi-square test

For subgroup analysis according to molecular tumor subtypes, patients were categorized into four groups: luminal A like (hormone receptor positive, human epidermal growth factor receptor 2 (HER2) negative, G1/G2), luminal B like (hormone receptor positive, HER2 negative, G3), HER2 positive (all HER2-positive tumors) and triple negative (hormone receptor negative, HER2 negative) [10, 11]. Due to missing values for hormone receptor and/or HER2 status, molecular subtypes could be determined for only 3670 of the 3754 randomized patients (Table 1).

### Data analysis

Descriptive statistics for the categorical data are provided in terms of absolute and relative frequencies. The non-normally distributed continuous variable age is described by medians and ranges. Associations between BMI group and patient or tumor characteristics were evaluated using the Kruskal-Wallis H test for age, the Mantel-Haenszel linear-by-linear association chi-square test for trends for the ordered categorical variables tumor size, nodal stage and grading, and the chi-square test for all other categorical variables.

Patient outcomes were analyzed in terms of both disease-free survival (DFS; with local, contralateral and distant disease recurrence as well as secondary primary tumors and death from any cause defined as the event) and overall survival (OS; with death from any cause defined as the event). Time-to-event data (median follow-up 65 months, range 1–96 months) were analyzed using the Kaplan-Meier method and summarized using medians, and 95 % confidence intervals, and survival curves for different BMI groups were compared using log-rank tests. All time-to-event intervals were measured from time of the primary diagnosis to date of the event or date of the last adequate follow-up in case no event was reported. To evaluate whether BMI constitutes an independent prognostic factor, we used Cox proportional hazards regression models adjusted for age (continuous), tumor size (pT1, pT2, pT3, pT4), nodal stage (pN0, pN1, pN2, pN3), tumor grade (G1, G2, G3), histological type (ductal, lobular, other), hormone receptor status (positive, negative), HER2 status (positive, negative), menopausal status (premenopausal, postmenopausal), type of surgery (breast conserving, mastectomy), chemotherapy treatment (FEC-Doc, FEC-DocG), antihormone therapy (yes, no) and undertreatment (fewer than 6 cycles vs. 6 cycles chemotherapy received). The BMI group underweight/normal weight was used as the reference group for the calculations of hazard ratios (HR) and 95 % confidence intervals (CI).

Statistical analyses were performed using IBM® SPSS® Statistics, version 21 (IBM Corp., Armonk, NY, USA). All statistical tests were two-sided, and *p* values of less than 0.05 were considered significant (no adjustment of significance levels for multiple testing).

## Results

### Associations of BMI with patient and tumor characteristics

Underweight/normal weight patients were younger on average (median age 50 years) and more often premenopausal (53 %) compared with overweight or obese patients (median age 54 years or older; fewer than 34 % premenopausal), and high BMI was associated with larger tumors and increased nodal involvement (Table 1). Patients with high BMI were also more likely to receive fewer than 6 cycles of chemotherapy, i.e., to be undertreated. In addition, severely obese patients (BMI ≥ 40.0 kg/m^2^) more often had hormone receptor-negative tumors, fewer tumors of the luminal A subtype and more tumors of the triple-negative subtype compared to patients with BMI less than 40.0 kg/m^2^ (see Table 1).

### Effect of BMI on disease-free and overall survival

**Fig. 1:**
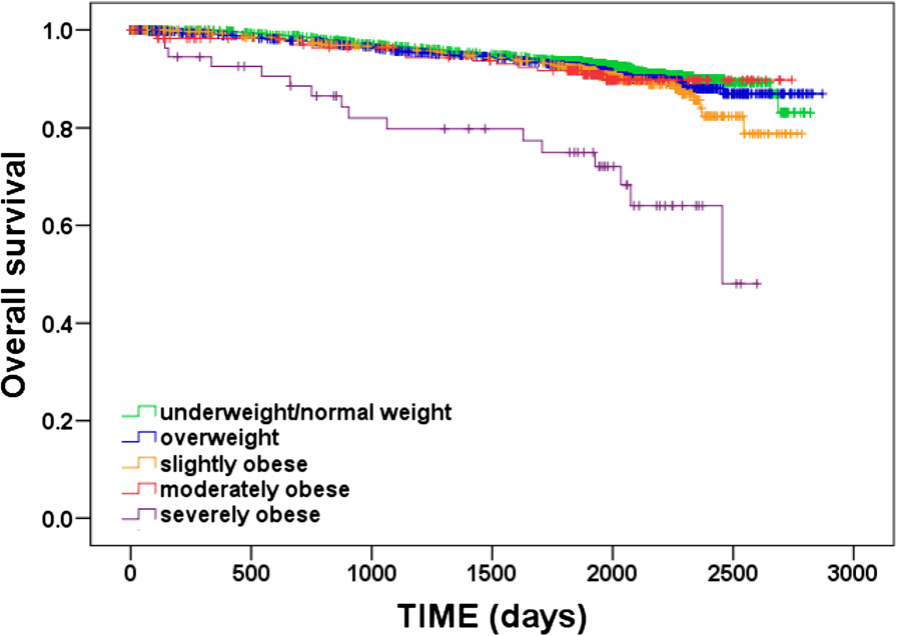
Kaplan–Meier plot of overall survival according to body mass index (BMI) group (underweight/normal: BMI < 25.0 kg/m^2^; overweight: BMI 25.0–29.9 kg/m^2^; slightly obese: BMI 30.0–34.9 kg/m^2^; moderately obese: BMI 35.0–39.9 kg/m^2^; severely obese: BMI ≥ 40.0 kg/m^2^)

Overall, 302 (8.0 %) patients died during the follow-up period: 120 (6.8 %) in the underweight/normal weight group, 99 (8.2 %) in the overweight group, 52 (9.4 %) in the slightly obese group, 15 (8.5 %) in the moderately obese group, and 16 (28.1 %) in the severely obese group. Univariate survival analyses revealed significant differences in OS among the BMI groups (log-rank test, *p* < 0.001; see Fig. 1). Pairwise comparisons using log-rank tests showed that OS differed significantly between severely obese patients and all other BMI groups (all *p* < 0.001), whereas there were no significant differences in OS among underweight/normal weight, overweight, slightly obese and moderately obese patients (all *p* > 0.05). Fully adjusted multivariate Cox regression analyses confirmed that BMI is a significant independent prognostic factor for OS (*p* = 0.005; Table 2). Overweight, slightly obese and moderately obese patients had no significantly different OS rates compared to underweight/normal weight patients (Table 2). However, severely obese patients showed significantly worse OS compared with underweight/normal weight patients (HR 2.79, 95 % CI 1.63–4.77, *p* < 0.001).

**Table 2:** Effect of body mass index (BMI) group (reference group underweight/normal weight) on overall survival (OS) and disease-free survival (DFS) in fully adjusted multivariate Cox regressions

Outcome parameter	Variable	Hazard ratio	95 % CI	*p* value
OS	BMI group			0.005
	*Overweight vs. underweight/normal weight*	1.00	0.75–1.32	0.979
	*Slightly obese vs. underweight/normal weight*	1.06	0.75–1.49	0.758
	*Moderately obese vs. underweight/normal weight*	1.07	0.62–1.86	0.799
	*Severely obese vs. underweight/normal weight*	2.79	1.63–4.77	<0.001
DFS	BMI group			0.001
	*Overweight vs. underweight/normal weight*	1.18	0.95–1.45	0.129
	*Slightly obese vs. underweight/normal weight*	1.06	0.81–1.40	0.664
	*Moderately obese vs. underweight/normal weight*	0.96	0.61–1.51	0.849
	*Severely obese vs. underweight/normal weight*	2.70	1.71–4.28	<0.001

*CI* confidence interval

**Fig. 2:**
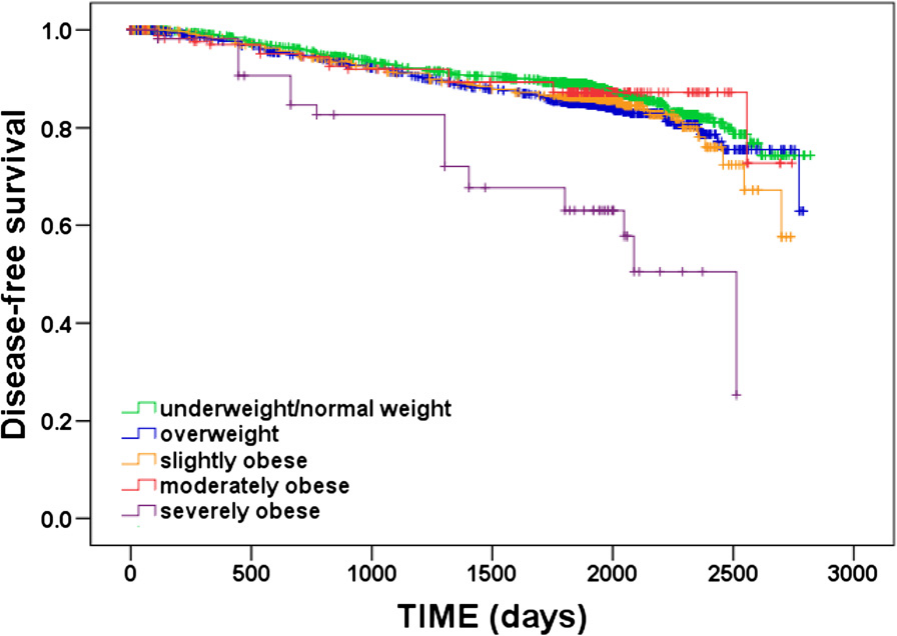
Kaplan–Meier plot of disease-free survival according to body mass index (BMI) group (underweight/normal: BMI < 25.0 kg/m^2^; overweight: BMI 25.0–29.9 kg/m^2^; slightly obese: BMI 30.0–34.9 kg/m^2^; moderately obese: BMI 35.0–39.9 kg/m^2^; severely obese: BMI ≥ 40.0 kg/m^2^)

In total, a recurrence during the follow-up period was observed in 502 (13.4 %) patients: 203 (11.5 %) in the underweight/normal weight group, 177 (14.7 %) in the overweight group, 80 (14.4 %) in the slightly obese group, 21 (11.9 %) in the moderately obese group, and 21 (36.8 %) in the severely obese group. Similar to the results for OS, univariate analyses showed significant differences in DFS among BMI groups (log-rank test, *p* < 0.001; Fig. 2), and pairwise comparisons revealed significant differences in DFS between severely obese patients and all other BMI groups (log-rank tests, all *p* < 0.001). Multivariate analyses confirmed the independent prognostic value of BMI for DFS (*p* = 0.001; Table 2), but again, only severely obese patients had significantly worse DFS compared with underweight/normal weight patients (HR 2.70, 95 % CI 1.71–4.28, *p* < 0.001).

### Subgroup analyses – effect of BMI on outcomes according to molecular tumor subtypes

**Fig. 3:**
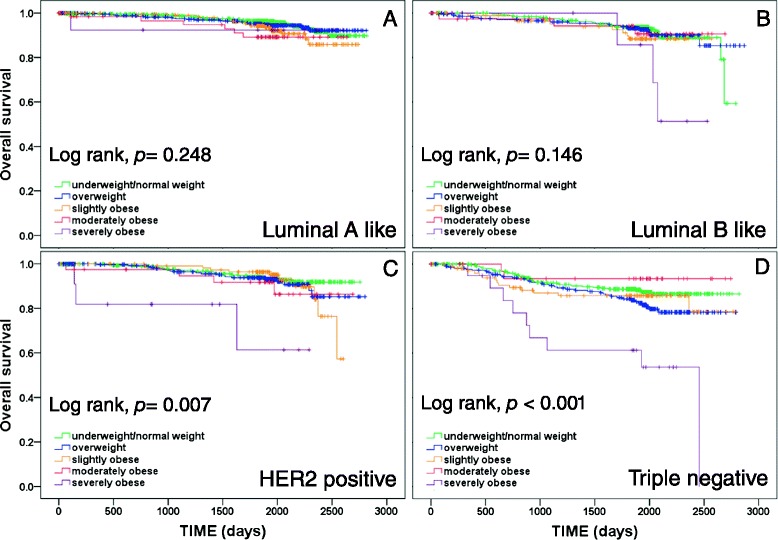
Kaplan–Meier plots of overall survival according to body mass index (BMI) group (underweight/normal, overweight, slightly obese, moderately obese, severely obese) in patients with tumor subtypes luminal A like (**a**), luminal B like (**b**), human epidermal growth factor receptor 2 (HER2) positive (**c**), and triple negative (**d**)

**Fig. 4:**
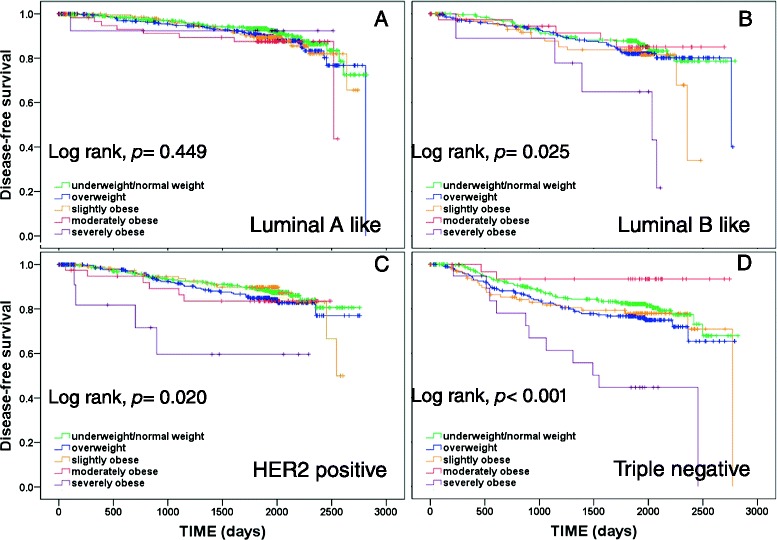
Kaplan–Meier plots of disease-free survival according to body mass index (BMI) group (underweight/normal, overweight, slightly obese, moderately obese, severely obese) in patients with tumor subtypes luminal A like (**a**), luminal B like (**b**), human epidermal growth factor receptor 2 (HER2) positive (**c**), and triple negative (**d**)

The results of the univariate survival analyses and survival plots for the different BMI groups according to the four molecular tumor subtypes are presented in Fig. 3 for OS and Fig. 4 for DFS. The effects of BMI group on OS and DFS as calculated based on fully adjusted multivariate Cox regressions are presented in Table 3.

**Table 3:** Effect of body mass index (BMI) group (reference group underweight/normal weight) on overall survival (OS) and disease-free survival (DFS) according to molecular subtype in fully adjusted multivariate Cox regressions

Molecular subtype	Outcome parameter		Hazard ratio	95 % CI	*p* value
Luminal A like	OS	BMI group			0.530
		*Overweight vs. underweight/normal weight*	0.86	0.48–1.52	0.594
		*Slightly obese vs. underweight/normal weight*	1.32	0.71–2.47	0.384
		*Moderately obese vs. underweight/normal weight*	1.72	0.69–4.30	0.247
		*Severely obese vs. underweight/normal weight*	1.51	0.20–11.42	0.688
	DFS	BMI group			0.851
		*Overweight vs. underweight/normal weight*	1.21	0.81–1.82	0.359
		*Slightly obese vs. underweight/normal weight*	1.10	0.66–1.83	0.714
		*Moderately obese vs. underweight/normal weight*	1.42	0.65–3.09	0.382
		*Severely obese vs. underweight/normal weight*	0.82	0.11–6.06	0.849
Luminal B like	OS	BMI group			0.384
		*Overweight vs. underweight/normal weight*	0.87	0.44–1.75	0.701
		*Slightly obese vs. underweight/normal weight*	1.29	0.55–3.00	0.561
		*Moderately obese vs. underweight/normal weight*	0.58	0.17–2.07	0.405
		*Severely obese vs. underweight/normal weight*	2.84	0.71–11.40	0.141
	DFS	BMI group			0.156
		*Overweight vs. underweight/normal weight*	1.18	0.72–1.93	0.513
		*Slightly obese vs. underweight/normal weight*	1.46	0.78–2.74	0.235
		*Moderately obese vs. underweight/normal weight*	0.76	0.29–2.00	0.584
		*Severely obese vs. underweight/normal weight*	3.32	1.17–9.46	0.024
HER2 positive	OS	BMI group			0.425
		*Overweight vs. underweight/normal weight*	0.86	0.46–1.62	0.646
		*Slightly obese vs. underweight/normal weight*	0.82	0.37–1.80	0.612
		*Moderately obese vs. underweight/normal weight*	1.36	0.43–4.35	0.601
		*Severely obese vs. underweight/normal weight*	2.78	0.75–10.34	0.128
	DFS	BMI group			0.198
		*Overweight vs. underweight/normal weight*	1.08	0.68–1.69	0.754
		*Slightly obese vs. underweight/normal weight*	0.81	0.44–1.50	0.503
		*Moderately obese vs. underweight/normal weight*	1.09	0.43–2.79	0.858
		*Severely obese vs. underweight/normal weight*	3.28	1.14–9.48	0.028
Triple negative	OS	BMI group			0.015
		*Overweight vs. underweight/normal weight*	1.41	0.88–2.25	0.155
		*Slightly obese vs. underweight/normal weight*	1.27	0.67–2.41	0.458
		*Moderately obese vs. underweight/normal weight*	0.51	0.12–2.21	0.368
		*Severely obese vs. underweight/normal weight*	3.85	1.69–8.77	0.001
	DFS	BMI group			0.011
		*Overweight vs. underweight/normal weight*	1.34	0.91–1.97	0.140
		*Slightly obese vs. underweight/normal weight*	1.29	0.77–2.17	0.335
		*Moderately obese vs. underweight/normal weight*	0.36	0.09–1.48	0.154
		*Severely obese vs. underweight/normal weight*	3.02	1.50–6.08	0.002

*CI* confidence interval, *HER2* human epidermal growth factor receptor 2

The lack of a significant effect of BMI group on survival in luminal A-like breast cancer as revealed by univariate log-rank tests was confirmed by fully adjusted multivariate Cox regressions, showing that BMI group had no significant independent prognostic value for OS or DFS (OS: *p* = 0.530; DFS: *p* = 0.851). Univariate log-rank tests showed significant differences among BMI groups with respect to DFS for patients with luminal B-like breast cancers, and with respect to both OS and DFS for patients with HER2-positive breast cancers. However, fully adjusted multivariate Cox regressions showed no independent effect of BMI group on OS or DFS in patients with luminal B-like or HER2-positive breast cancers (see Table 3). Consistent significant effects of BMI group on outcomes in both univariate and multivariate analyses were found only for patients with triple-negative breast cancer (TNBC). Univariate analyses showed that OS and DFS differed significantly among BMI groups and multivariate analyses confirmed the independent prognostic effect of BMI group on OS (*p* = 0.015) and DFS (*p* = 0.011). However, similar to the results for the full data set, the effect of BMI in TNBC was only apparent for severely obese patients, who had significantly worse OS and DFS compared to underweight/normal weight patients, while no significant hazard ratios were observed for overweight, slightly obese or moderately obese patients compared with underweight/normal weight patients (see Table 3).

## Discussion

This retrospective analysis of data collected in the randomized controlled SUCCESS A trial shows significantly worse outcomes (OS, DFS) in severely obese patients (BMI ≥ 40 kg/m^2^), but not in moderately obese (BMI 35.0–39.9 kg/m^2^), slightly obese (BMI 30.0–34.9 kg/m^2^), or overweight (BMI 25.0–29.9 kg/m^2^) patients compared with underweight/normal weight BMI < 25.0 kg/m^2^) patients with early breast cancer.

The overall negative effect of obesity on outcomes in breast cancer patients is well known and has recently been confirmed by two large meta-analyses [3, 12]. However, heterogeneous patient samples, different statistical approaches and the fact that the obesity and reference groups were not consistently defined in the included studies hamper a more detailed analysis of the association between obesity and outcome with regard to the extent of the negative effect associated with different obesity levels. Large single clinical trials are better suited to evaluate the effect of BMI on outcomes for defined breast cancer patient cohorts and treatment regimens. Retrospective analyses of 2887 node-positive breast cancer patients enrolled in the randomized phase III trial BIG 02–98 and of 1310 node-positive high-risk breast cancer patients enrolled in the randomized phase III ADEBAR trial showed worse DFS and OS rates for obese (BMI ≥ 30) compared with nonobese (BMI < 30) women [5, 13]. However, to our knowledge, our study is the first analysis of a large homogenous patient sample from a prospective randomized clinical phase III trial with modern adjuvant chemotherapy (anthracycline- and taxane-based) that investigated the impact of different stages of obesity on breast cancer outcomes. Our results demonstrate that analyses that are based solely on comparing obese (BMI ≥ 30) with nonobese (BMI < 30) patients might not be sufficient to evaluate the effect of obesity on breast cancer outcome, as we found a significant effect only for severe obesity, but not for moderate or slight obesity.

In our study, analysis of the different subgroups of breast cancer revealed no significant impact of obesity on outcomes in the luminal A-like, luminal B-like and HER2-positive subtypes, but significantly poorer DFS and OS in severely obese compared with underweight/normal weight patients with triple-negative breast cancer.

The recently published secondary analysis of the Women’s Health Initiative (WHI) randomized clinical trials demonstrated that for women older than 50 years obesity grade 2 or 3 (i.e., BMI > 35.0) was strongly associated with the risk for hormone receptor-positive breast cancer [14]. Thus, it seems surprising that we did not find a significant effect of obesity on survival in patients with breast cancer of the hormone receptor-positive luminal A and luminal B subtype. However, generally the data regarding the effect of BMI on survival in different breast cancer subtypes are quite heterogeneous and inconsistent. A meta-analysis of 21 studies comprising more than 80,000 patients yielded no evidence that the association of obesity with breast cancer outcomes differs by hormone receptor status [15]. Similarly, Pajares and coauthors, who found significantly poorer outcomes for patients with BMI ≥ 35 compared to patients with BMI < 25, stated that the magnitude of the effect was similar across different subtypes (estrogen receptor (ER)/progesterone (PR) positive and HER2 negative; HER2 positive; triple negative) [7]. In contrast, an analysis of pooled data from three adjuvant trials of the Eastern Cooperative Oncology Group with anthracycline-based chemotherapies in 6885 patients revealed significantly poorer outcomes for obese patients (BMI ≥ 30) than for nonobese patients with hormone receptor-positive disease, but no negative impact of obesity on survival in patients with other breast cancer subtypes [6]. A recent pooled analysis evaluated the impact of BMI on DFS and OS according to breast cancer subtypes in 8872 patients with primary breast cancer treated with neoadjuvant chemotherapy [16]. The authors found that overall obese (BMI 30 to < 40) and very obese (BMI ≥ 40) patients had shorter DFS and OS than normal weight (BMI 18.5 to < 25) patients, and subgroup analyses revealed consistent negative effects of BMI on survival in luminal-like (ER/PR-positive and HER2-negative) and TNBC but not in HER2-positive tumors. In our study, the DFS and OS hazard ratios for severe obesity (reference group underweight/normal weight) were similar for the luminal B-like, HER2-positive and triple-negative subtypes (range 2.78–3.85), indicating that the lack of significance in the luminal B-like and HER2- positive subgroups found in our study might be attributable to the smaller number of severely obese patients and the resulting reduced statistical power. In contrast, DFS and OS hazard ratios for severe obesity were much lower in the luminal A-like subgroup (1.51 and 0.82, respectively), suggesting that even severe obesity does not constitute an additional risk factor for patients with low-risk breast cancer of the luminal A-like subtype. We are not aware of any other study that evaluated the effect of obesity on survival in breast cancer subtypes and distinguished between luminal A and luminal B tumors. It might be speculated that an increased risk in severely obese patients with hormone receptor-positive tumors could have been masked by anti-hormonal therapy in our study, in particular in the group of patients with low-risk luminal A-type breast cancer. The fact that the analysis of data gained in the Eastern Cooperative Oncology Group adjuvant trial E1199 showed a strong negative effect of obesity on outcome in patients with hormone receptor-positive breast cancer that received a similar endocrine treatment as the patients in our study (i.e., either tamoxifen alone or tamoxifen followed by an aromatase inhibitor) [6] does not argue against such a possible masking effect of antihormone therapy, as the median follow-up time in the E1199 trial was 95 months and thus far exceeded the duration of anti-hormone therapy. Taken together, the conflicting data on the effect of obesity on survival in different subtypes of breast cancer indicate that the mechanistic link between obesity and breast cancer survival is complex and not yet fully understood.

Obesity is known to be often accompanied by cardiovascular risk factors such as glucose and lipid disorders and hypertension, and the adverse effect of high BMI on long-term outcome is likely mediated at least partly through these obesity-related metabolic abnormalities. A recent meta-analysis showed that even normal-weight individuals were at an increased risk for all-cause mortality and cardiovascular events if they were metabolically unhealthy, while – on the other hand – obese individuals had an increased risk even if they were metabolically healthy when only studies with a follow-up of at least 10 years were analyzed [17]. Another large recent meta-analysis found that the presence of metabolic abnormalities (i.e., metabolic syndrome) is associated with an increased breast cancer risk in adult women [18]. These results point to a link between metabolic syndrome, obesity and breast cancer and suggest that the presence of metabolic abnormalities should be included in future studies evaluating the effect of obesity on survival in breast cancer patients.

A strength of this study is the fact that the analysis is based on a homogenous patient sample from a large prospective randomized clinical trial, which reduces the potentially confounding effects of heterogeneous patient samples and different treatment regimens. In addition, this is the first study in which the effects of BMI on survival in breast cancer patients were evaluated separately for the four biological tumor subtypes using three distinct classes of obesity. Limitations of the study are the lack of information on Ki-67, needed for more accurate subdivision of different breast cancer subtypes, and the low number of patients with severe obesity (leading to limited statistical power in particular regarding the subgroup analyses with respect to the four tumor subtypes). In addition, no data on metabolic abnormalities (metabolic syndrome) were available. Furthermore, it is unknown whether the reduced survival observed in severely obese breast cancer patients is a direct consequence of obesity itself (as, for example, the increased chemotherapy doses might partly diffuse into fat tissue and thus not fully reach the tumor) or rather caused by obesity-related comorbidities.

## Conclusions

In conclusion, this single-study analysis of 3754 patients demonstrated that only severe obesity (BMI ≥ 40) is associated with worse outcomes in operable high-risk early breast cancer, especially in the triple-negative subtype. More studies are needed to evaluate the effect of obesity on outcomes in breast cancer patients with respect to both the obesity level that constitutes an independent risk factor for reduced survival, and the effect of obesity in different tumor subtypes.

## Abbreviations

BfArM: German Federal Institute for Drugs and Medical Devices
BMI: body mass index
CI: confidence interval
DFS: disease-free survival
ER: estrogen receptor
FEC-Doc: 5-fluorouracile, epirubicine, cyclophosphamide – docetaxel
FEC-DocG: 5-fluorouracile, epirubicine, cyclophosphamide – docetaxel, gemcitabine
GCP: good clinical practice
HER2: human epidermal growth factor receptor 2
HR: hazard ratio
OS: overall survival
PR: progesterone receptor
TNBC: triple-negative breast cancer
